# The Adaptation, Implementation, and Performance Evaluation of Intake24, a Digital 24-h Dietary Recall Tool for South Asian Populations: The South Asia Biobank

**DOI:** 10.1016/j.cdnut.2025.104543

**Published:** 2025-01-16

**Authors:** Divya Bhagtani, Birdem Amoutzopoulos, Toni Steer, David Collins, Suzanna Abraham, Bridget A Holmes, Baldeesh K Rai, Rajendra Pradeepa, Sara Mahmood, Abu Ahmed Shamim, Poorvee Mathur, Lathika Athauda, Laksara De Silva, Khadija I Khawaja, Vinitaa Jha, Anuradhani Kasturiratne, Prasad Katulanda, Malay K Mridha, Ranjit M Anjana, John C Chambers, Polly Page, Nita G Forouhi

**Affiliations:** 1 University of Cambridge, MRC Epidemiology Unit, Institute of Metabolic Science, Cambridge, United Kingdom; 2 Department of Epidemiology and Biostatistics, School of Public Health, Imperial College London, London, United Kingdom; 3 Madras Diabetes Research Foundation, Chennai, Tamil Nadu, India; 4 Department of Endocrinology & Metabolism, Services Institute of Medical Sciences, Services Hospital, Lahore, Pakistan; 5 Centre for Non-Communicable Diseases and Nutrition (CNCDN), BRAC James P Grant of Public Health, BRAC University, Dhaka, Bangladesh; 6 Office of Research, Max Super Speciality Hospital (Devki Devi Foundation), New Delhi, India; 7 Department of Public Health, Faculty of Medicine, University of Kelaniya, Ragama, Sri Lanka; 8 Department of Clinical Medicine, Faculty of Medicine, University of Colombo, Colombo, Sri Lanka; 9 Lee Kong Chian School of Medicine, Nanyang Technological University, Singapore, Singapore

**Keywords:** diet, Intake24, dietary assessment, 24-h dietary recall, South Asia

## Abstract

**Background:**

South Asia’s diverse food supply, food preparations, and eating behaviors require dietary instruments that reflect the consumption patterns of South Asians to enable context specific dietary assessment. Such instruments are not readily available for detailed dietary assessment at scale in South Asia.

**Objectives:**

We describe the adaptation, implementation, and performance evaluation of Intake24, an open-source digital 24-h dietary recall tool, for dietary assessment in South Asia.

**Methods:**

We adapted Intake24 for dietary assessment in the South Asia Biobank (SAB), a large population-based study in Bangladesh, India, Pakistan, and Sri Lanka. Intake24 adaptation encompassed the development of a South Asian food database with commonly consumed foods, linked with corresponding portion sizes, food probes, and nutrient information. Trained interviewers conducted the 24-h recalls. Performance of Intake24 was evaluated in 29,113 South Asian adults.

**Results:**

The South Asia Intake24 food database included 2283 items and demonstrated good coverage of foods consumed across SAB regions. Median recall completion time was 13 min. Quality control metrics showed 99% of recalls included >8 items and 8% had missing foods. Median energy intake was higher in younger individuals compared to older, and in males compared to females. Underweight participants reported lower energy intake, with no discernible difference across other BMI categories.

**Conclusions:**

Intake24 enables comprehensive dietary assessment in regions of South Asia and will facilitate the analysis of dietary patterns, food and nutrient intake, and their relationship with health outcomes among South Asians.

## Introduction

Suboptimal diet is a leading risk factor for noncommunicable diseases such as type 2 diabetes and cardiovascular disease, with a disproportionately high burden in South Asian countries [1]. Despite diet being a key modifiable risk factor, dietary assessment is seldom included in research. This is particularly true in low-income and middle-income countries (LMICs) where a lack of appropriate dietary assessment tools limit large-scale dietary data collection [2,3]. Assessing and monitoring malnutrition in all its forms, including energy intake and diet quality, in LMICs, necessitates the collection of high-quality individual-level dietary data. Detailed dietary data collection is also essential for assessing nutritional status given that the relationship between food consumption and nutritional status is close knit [4].

There is a paucity of dietary intake data in many LMICs because of factors such as high cost and time burden of collecting detailed quantitative dietary data, as well as limited investment in research infrastructure, including resources and technology for efficient data collection, coding, and processing [2,5]. Recent technological advances and the development of web-based and computer-based dietary assessment systems have substantially reduced the need for manual data entry and coding and have enabled large-scale dietary data collection that is more cost and resource efficient and less burdensome for researchers and research participants [2]. However, despite the recent growth in availability of such systems, few have reported the methodological approaches in developing dietary data collection systems, particularly with regard to their contextual adaptation, implementation, and evaluation in LMICs [[5], [6], [7], [8], [9], [10], [11]]. Contextual adaptation is essential to appropriately capture dietary intake across diverse settings, especially in LMICs, where food supplies, meal composition, preparation methods, and cultural practices differ from those in high-income countries. Without localization, dietary assessment systems risk face validity and inaccuracies that can misrepresent dietary patterns. Tailoring such tools to reflect local foods, cooking methods, and portion sizes is important for improving data quality and increasing participant engagement.

The 24-h dietary recall method has long been used in dietary surveillance and has gained interest in epidemiologic studies due to its ability to provide detailed individual-level intake of all food and drinks consumed rather than relying on a fixed list of items that allow ranking of participants. For comparing dietary intakes across countries or regions, the 24-h recall method facilitates the collection of culturally specific dietary data, capturing granular information about specific foods that may be more relevant within cultural contexts rather than providing generic examples of food groups [12]. In this study, we describe the adaptation, implementation, and evaluation of a digital 24-h dietary recall tool—**Intake24**, for interviewer-led administration in 4 South Asian countries included in the South Asia Biobank (SAB), a large population-based study in Bangladesh, India, Pakistan, and Sri Lanka [13]. Intake24 is an open-source web-based dietary assessment system, based on the multiple pass 24-h recall method to measure individual-level food and drink intake during the previous day [[14], [15], [16]]. Our objective was to adapt and implement this tool for interviewer-led dietary assessment in the SAB.

## Methods

### Study setting, population, and participant assessments

In brief, the SAB established a cohort of healthy South Asian men and women aged 18 y and above from 118 surveillance sites in 5 regions: Bangladesh, North India, South India, Pakistan, and Sri Lanka. Detailed information about the SAB research sites, recruitment, and assessments have been reported previously [13]. Participants gave written informed consent and attended the survey sites where they provided biological samples (blood and spot urine), completed a health and lifestyle questionnaire, underwent physical measurements, and participated in physical activity monitoring. The SAB data collection started in November 2018 and dietary assessment using Intake24 for collection of a single 24-h dietary recall was introduced in January 2020. Intake24 was administered using a standardized protocol by trained interviewers. This study was conducted according to the guidelines laid down in the Declaration of Helsinki. Research approval was obtained from the Imperial College London Ethics Committee (ref: 18IC4698) and the local institutional review boards in each of the participating countries.

**FIGURE 1:**
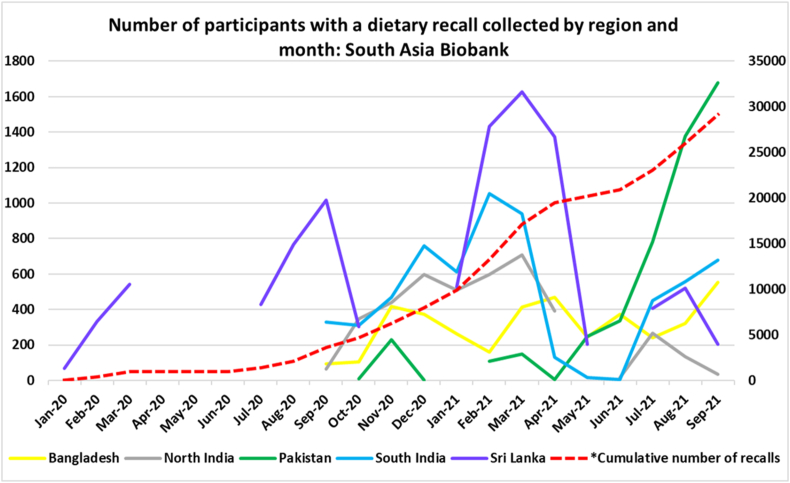
Number of participants with a dietary recall collected between January 2020 and September 2021 by region and month of dietary data collection in the South Asia Biobank. ∗Cumulative number of recalls are based on the right vertical axis ranging from 0 to 35,000. Participants with sex other were excluded due to small number (*n* = 17).

Data collection in the SAB paused and restarted through the disruptions caused by the COVID-19 pandemic, working within the region-specific government rules and adapting to the local circumstances as appropriate. Figure 1 shows the number of participants with a dietary recall by region and month of data collection.

### Adaptation of Intake24 for dietary assessment in South Asia

**FIGURE 2:**
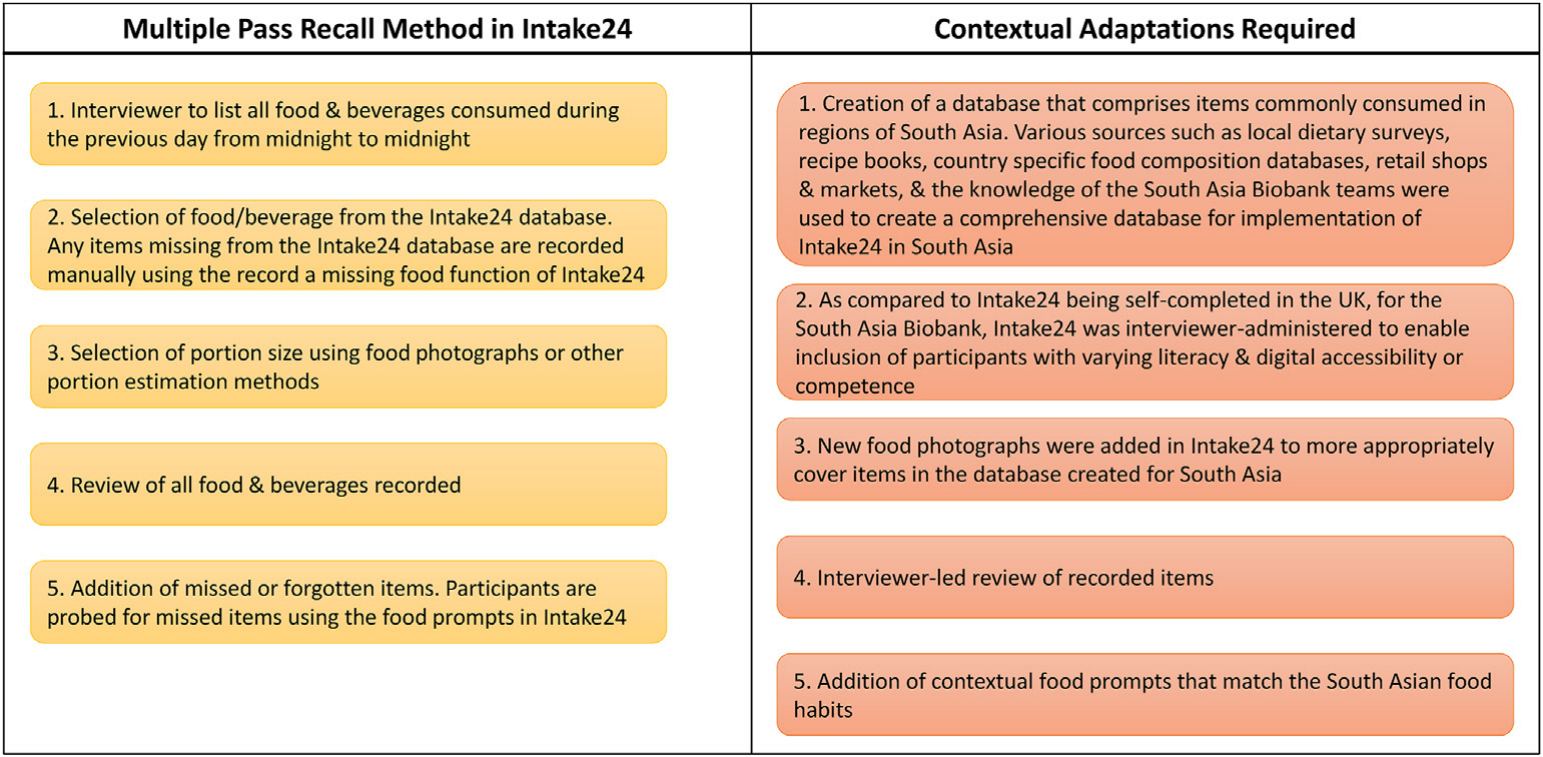
Illustration of the multiple pass recall method used in Intake24 and key contextual adaptations for implementation in the South Asia Biobank.

Intake24 is based on the multiple pass recall method that guides individuals to recall everything they consumed over the previous 24-h period (from midnight to midnight). Through the recall question structure, individuals are directed to first list all food and drink items consumed (in each eating occasion), followed by recording quantities and further information corresponding to each food and drink recorded. Figure 2 illustrates the multiple pass recall method in Intake24 and the key contextual adaptations for implementation in the SAB. This included a South Asia–orientated food list with matched nutrient composition codes and customization of the recall questions to better suit the study and regional context. Originally developed at Newcastle University, United Kingdom, as a self-completion tool [14,16,17], Intake24 was adapted for the SAB to facilitate interviewer-administered data collection to enable inclusion of participants with varying literacy and digital accessibility or competence. Initially designed and validated (against interviewer-led recalls) for participants aged 11–24 y, Intake24 has undergone subsequent evaluation for criterion validity using the doubly labeled water method in adults aged 40–65 y [16,17].

**FIGURE 3:**
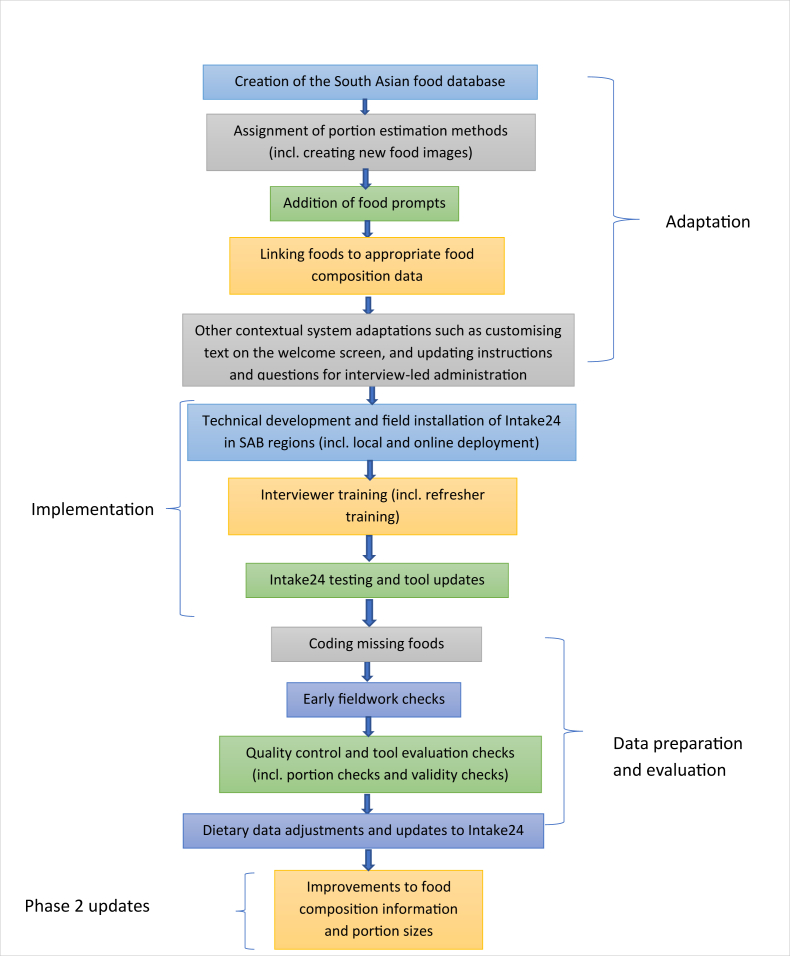
Flowchart of steps involved in the adaptation, implementation, and data preparation and performance evaluation of Intake24 for dietary assessment in the South Asia Biobank.

We pragmatically and swiftly adapted Intake24 to facilitate dietary data collection in the SAB, a process undertaken while participant recruitment was already underway in SAB. Simultaneously, we continued to iteratively refine and improve the Intake24 tool in parallel with active dietary data collection (see Food composition database section). Adaptation of Intake24 for the SAB included the following key steps: creation of a South Asian food database, development of locally relevant food portion photographs and allocation of portion size estimation methods, addition of food accompaniment prompts (to support capture of foods commonly consumed together), refinement of the provision to record missing foods, and linking of food composition codes to all items in the food database for automated calculation of energy and nutrient intakes. This was followed by technical development and field implementation of the Intake24 interface on data collection tablets for facilitating local and online deployment in all SAB regional sites (including where internet availability was poor or unreliable). The flowchart in Figure 3 provides an overview of Intake24 adaptation, implementation, data preparation and evaluation, and further planned work. Tool enhancements including but not limited to improving food composition information and portion size estimation for South Asia are ongoing.

### Identification of foods for the South Asian food database

To facilitate adaptation of Intake24 and creation of a comprehensive food database for regions of South Asia, the UK Intake24 food database was used as the starting point. A standardized protocol was developed for reviewing food and drink items in the UK Intake24 food database to determine those to be retained, deleted, or added to create the South Asian Intake24 food database. This was carried out in collaboration with each of the SAB regional research teams including local nutrition experts. Following the International Network of Food Data Systems guidelines for describing foods [18], regional teams provided information including food description, local names, brand names, relevant portion estimation methods, and food group for each new item to be added to the Intake24 South Asian food database. Regional teams used sources such as local dietary surveys, recipe books, food composition databases (FCDBs), retail shops and markets, and local knowledge for identification of food items. Where appropriate, regional teams provided local names and translations in their regional language, which were phonetically entered into the food database in English.

### Portion size estimation methods

**FIGURE 4:**
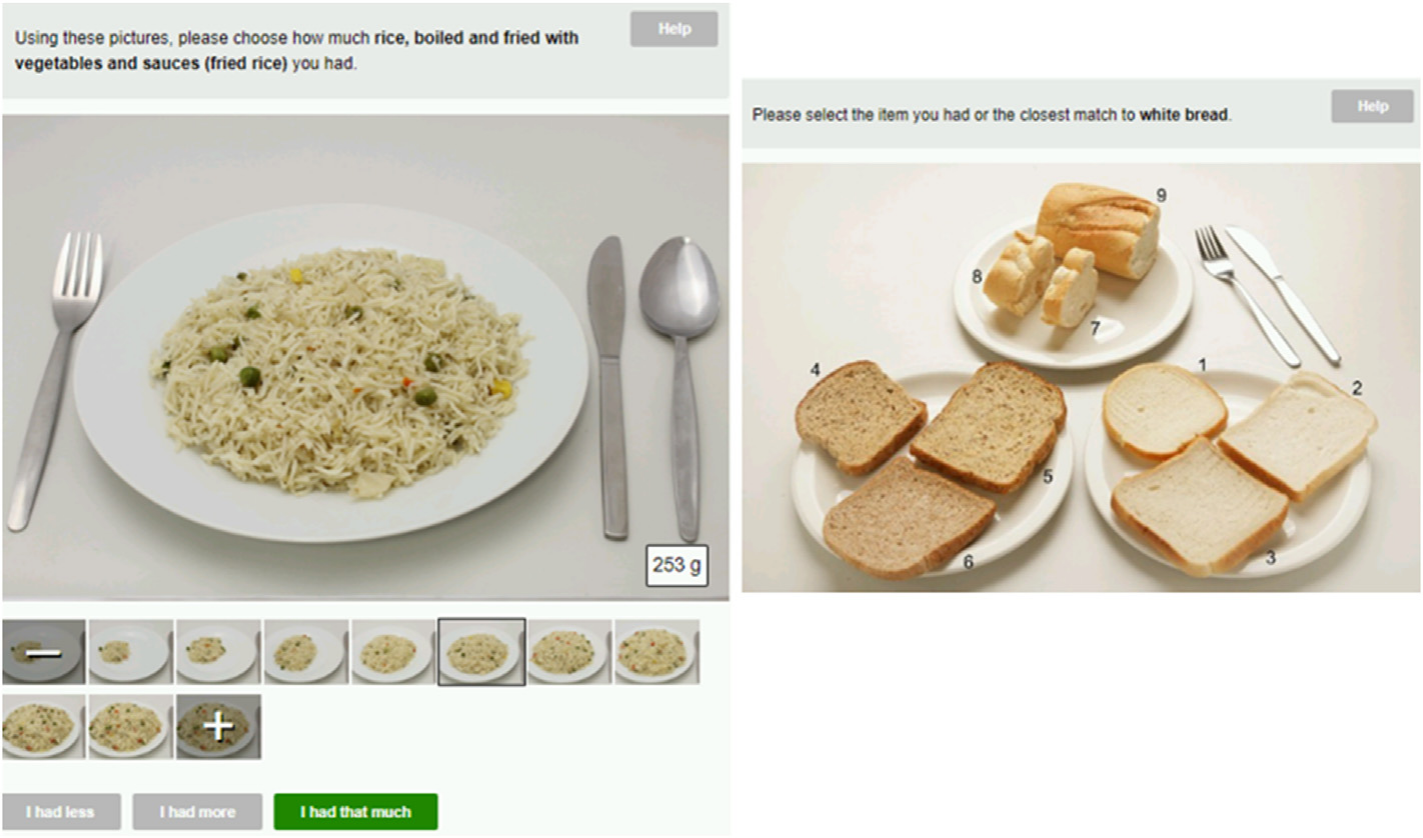
Illustrative examples of portion images in the South Asia version of Intake24. Left image: fried rice (as served); right image: white bread (guide).

Intake24 offers 4 different ways to estimate portion size: as-served images, guide images, sliding scale for drinks, and standard portions [14]. As-served images are generally a series of 7 photographs showing progressively increasing quantities of foods, whereas guide images depict standardized portion items, for example, slices of bread, in various predetermined portion sizes (Figure 4). After review, we determined that 120 as-served and 79 guide images from the UK Intake24 food database were appropriate for inclusion in the South Asian Intake24 database. A total of 17 new image sets (13 as-served and 4 guide) relevant to South Asian foods not well represented in the UK Intake24 food portion image database were photographed using standard guidelines [19]. These new images were added to cover a wide range of commonly consumed South Asian foods including curry (both wet and dry consistency), rice-based dishes, chapatti and other cereal-based breads, raita (yogurt based), chutney, pickle, and fritters. Portion weights for new images were aligned with those existing within the UK Intake24 with an exception for rice where the portion range was taken from the food portion size atlas [20] to cover larger servings of rice consumed in some regions of South Asia. Consistent with the approach generally taken in Intake24, foods with similar appearance and or consistency were assigned the same food images for efficiency of tool development.

Leveraging insights from the SAB regional teams, it became apparent that meat-based and seafood-based dishes in regions of South Asia are typically consumed in smaller quantities than that in the United Kingdom, whereas rice, a regional staple, is commonly eaten in larger portions. Consequently, we adapted portion images by including 9 as-served images, that is, with an additional 2 smaller portions for all meat and seafood-based dishes and 2 larger portions for rice-based dishes. Moreover, Intake24 includes a “±” key, allowing users to record portion sizes that are either larger or smaller than the as-served images. As curries and rice-based dishes constitute key components of daily diets in South Asia, we conducted an evaluation of portion sizes for these foods to ensure South Asian relevance, using a literature review before deploying Intake24. This review aimed to identify publications that quantify typical consumption patterns and portion sizes for these dishes within South Asian populations. Our findings indicated that the portion size range in the South Asia version of Intake24 aligned with those in regions of South Asia and offered a broader selection [[21], [22], [23]].

Standard portions in Intake24 include household measures (teaspoons and tablespoons) and unit weight descriptions such as medium apple and small flatbread. These were based on the UK Food Portion Size book [24], regionally relevant published literature, online searching, or by weighing foods.

### Food accompaniments

Intake24 prompts for accompanying food items such as milk in tea or butter on toast, which can be commonly forgotten in dietary reporting. We adapted and expanded the existing food prompts in the UK Intake24 to match South Asian food habits by including contextual prompts such as “Did you have any pickle or chutney with that?” or “Did you sweeten your tea (e.g. sugar, jaggery, sweetener)?.”

### Missing foods

We adapted Intake24’s missing foods feature for interviewers to report a food, dish, or drink if they were unable to find it in the tool [14]. On selecting the “report a missing food” option, the system allows entry of details on the missing food’s name, brand or place of purchase, description, cooking method, and quantity consumed. For SAB, the missing food questions were adapted to capture information better suited to an interviewer-led format, focusing on cooking method and actual quantity consumed, rather than quantity served and leftover, to reflect the diverse cooking practices in South Asia. Food and drink items included within the Intake24 food database are autocoded to generate corresponding energy and nutrient intakes; however, in contrast, for missing foods, manual coding is required.

### Food composition database

We undertook a review and evaluation of the existing and available FCDBs from each SAB country [[25], [26], [27]]. This review aimed to assess each FCDB for content and quality by devising a scoring system to prioritize the available resources for use. Each FCDB was scored between 0 and 15, with a maximum quality assessment score of 3 for each of the following criteria: *1*) source of data (analytical compared with calculation methods), *2*) age of data, *3*) coverage of foods, *4*) coverage of nutrients, and *5*) ease of use. A score of 1 indicated low quality. The Indian FCDB had the highest quality assessment score of 13, followed by the Bangladeshi and Pakistani FCDB with a score of 10 and 9, respectively. A Sri Lankan FCDB was not available at the time of our evaluation. The number of items covered by the FCDBs varied from 528 items in the Indian FCDB to 381 and 215 items in the Bangladeshi and Pakistani FCDB, respectively. The Indian and Pakistani FCDB values are based on analytical methods, whereas the Bangladeshi FCDB values are based on calculation methods applied using existing data sources [[25], [26], [27]]. Despite having the highest assessment score, the Indian FCDB was inadequate to use without further work because it did not include composition values for cooked foods (except for egg). Individually and collectively, the existing country-specific FCDB varied substantially in the coverage of foods and nutrients, included out of date or missing information for some foods and nutrients, and overall did not provide sufficiently comprehensive or readily available source of nutrient information for the South Asia food database created for Intake24. Therefore, we took the decision to assign nutrient composition information for South Asia using nutrient composition values from the UK Nutrient Databank (NDB) comprising ∼10,000 food composition codes [28]. Two researchers (DB and SA) matched and assigned food items to the UK NDB codes using a comprehensive approach that considered factors such as local food description, food groups, scientific food name, and botanical classification. In instances where a particular food or ingredient was not available in the UK NDB, a similar alternative item was selected. This initial coding was then reviewed, discrepancies were discussed, investigated in order to resolve, and the coding was verified by senior researchers (BAH, TS, and BA) to ensure suitability. We adopted this method to deliver the Intake24 tool for South Asia while using a phased approach that allowed dietary data capture from participants being recruited into the ongoing study which began in 2018, with plans to return to improvements in the food composition work at a later phase of the project.

### Implementation of Intake24 in SAB

#### Technical development and field installation

Intake24 was completed by interviewers using Tablets, accessed via the SAB data collection platform [13]. Owing to intermittent internet connectivity in some SAB regional sites, the Intake24 application was installed on local servers accessed by Wi-Fi at each study site. Where internet availability permitted, Intake24 was also used online (https://ndns.intake24.org/surveys/demo-sab?genUser) hosted from the United Kingdom (Cambridge). This dual protocol of local servers and online access was extended following the COVID-19 pandemic to maximize flexibility for data collection.

#### Interviewer training

Training sessions were conducted for each regional team to ensure standardized approaches were used to collect 24-h dietary recall data. In Sri Lanka, North India, and South India, training sessions were conducted locally, in-person, and comprised 2 half-day sessions. These included an explanation of the 24-h dietary recall method, demonstration of Intake24 features, hands-on practice sessions, and detailed feedback on practice recalls completed by interviewers both before and after the training. For Bangladesh and Pakistan, in-person training was not feasible due to COVID-19 restrictions. Instead, virtual training sessions were organized via Zoom (Zoom Video Communications; Qumu Corporation). Despite the disruptions caused by the pandemic, all regional teams were provided with ongoing support, including refresher training, additional practice exercises, and performance feedback before data collection resumed post local COVID-lockdown. Regional teams were also supplied with comprehensive written materials addressing practical issues related to the use of Intake24 and general dietary data collection practices. During the early stages of dietary data collection in each SAB region, recall data were reviewed by the Cambridge and Imperial College London research teams. This process involved a detailed review of a subset of recalls, performance evaluation checks (as described in the next section), and investigation of any identified issues, following which feedback was provided for interviewers to improve data quality.

#### Analysis and evaluation of dietary data collected in the SAB for assessment of tool performance

Quality control (QC) checks were conducted by the research team using dietary recall data available from 29,113 participants at the time of this evaluation. To facilitate automated and regular QC checks, a Microsoft Access database was developed to enable dietary data to be efficiently viewed, managed, queried, and updated, if required. The frequency of checks was weekly during the initial 2 months of data collection and monthly thereafter. Specifically, QC checks included the following: %recalls that took <10, 5, and 2 min to complete (likely indicator of incomplete reporting); %recalls with <8 items (likely indicator of incomplete reporting); %missing foods (likely indicator of lack of regional food coverage in Intake24); %recalls with implausible estimated energy intake <500 kcal/d in females and <800 kcal/d in males (likely indicator of under-reporting as per widely applied criteria) [29,30], and %recalls with estimated energy intake >3500 kcal/d in females and >4200 kcal/d in males (likely indicator of over-reporting as per widely applied criteria) [29,30]. QC checks also included comparisons of reported energy intake by sex, age, and BMI. For BMI, South Asian specific categories were used: <18.5 kg/m^2^ indicating underweight; 18.5–22.9 kg/m^2^, normal weight; 23 kg/m^2^ or higher, overweight or obese [31].

Additionally, we conducted checks on reported portion sizes to identify and address any tool-related errors. For each of the 91 food groups in Intake24, we generated portion size box plots by region and sex to detect outliers. Outliers were defined as values outside the range of 3 times the IQR from the nearest quartile.

Results from QC checks were shared with each regional team along with comprehensive feedback on interviewer-collected dietary recall data, with provision of further training if indicated. The checks also informed tool enhancements, such as, but not limited to, addition of new regional foods, improvements to food names/descriptions, portion sizes, and updates to food composition information.

## Results

Following the processes to develop a regionally appropriate dietary assessment tool including food items commonly consumed in Bangladesh, India, Pakistan, and Sri Lanka, the South Asia version of Intake24 was created. This included 2283 foods with appropriate portion size estimation methods, food prompts, and energy and nutrient composition data.

**FIGURE 5:**
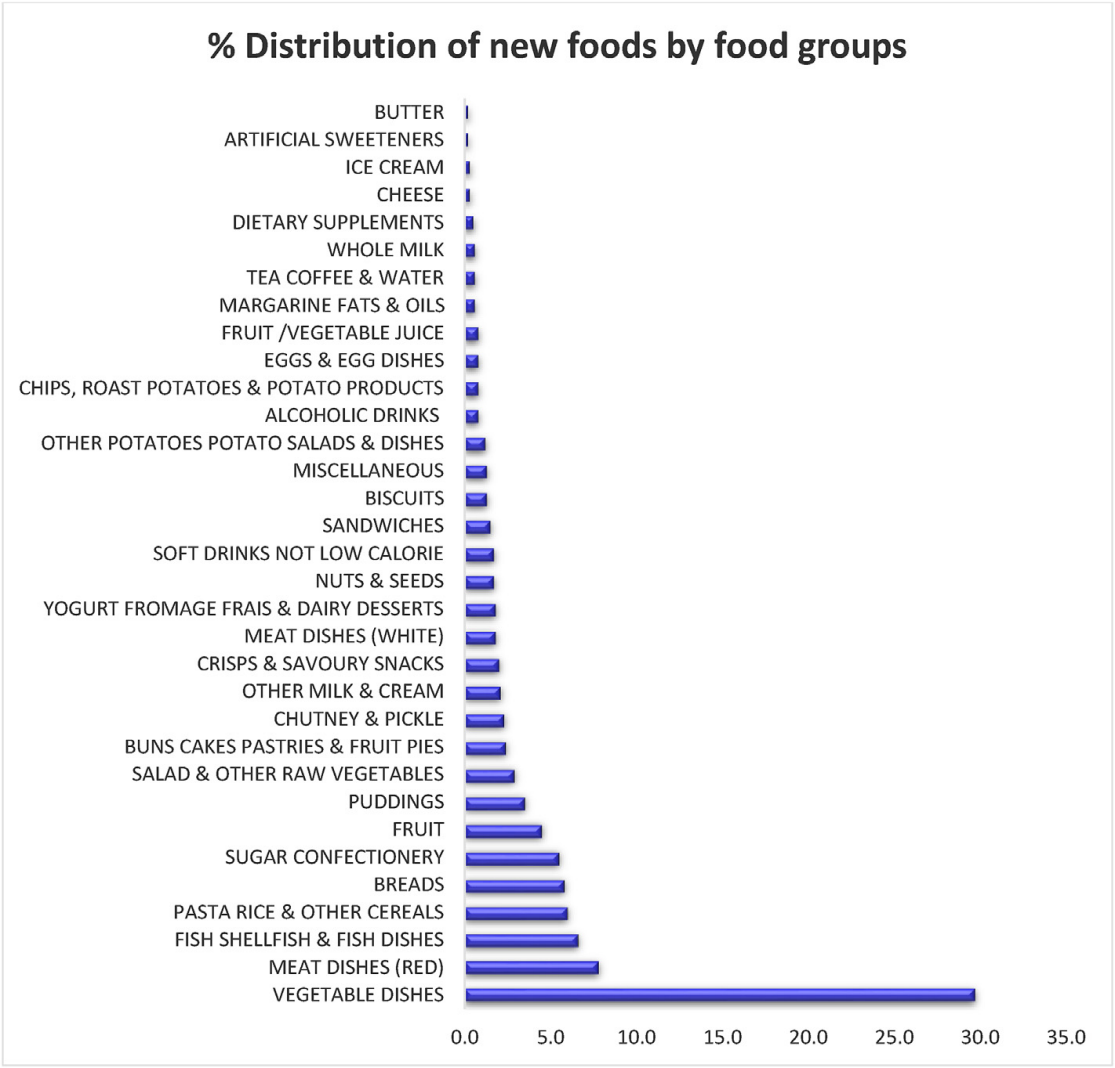
Food group distribution of new regional food items (*n* = 1152) added to the food database for the South Asia version of Intake24.

The UK Intake24 food database was used as the starting point for compiling the South Asian food database. Generic items such as common fruits, vegetables, and spices were retained from the UK Intake24 food database, whereas items that are typically British and unlikely to be consumed in South Asia such as beef Wellington, gammon rashers, or anchovies were deleted. A range of food items and mixed dishes commonly consumed in the study regions such as vegetable, meat, and fish-based curries, local snacks, sweets, breads, and drinks were added (Supplemental Table 1). With these adaptations, the South Asian food database comprised 1131 (49.6%) items that were retained from the UK Intake24 database and 1152 (50.4%) new South Asian foods and dishes. Figure 5 shows the distribution of types of new foods added to the South Asian food database. In summary, vegetable-based, meat-based, and fish-based dishes, pasta, rice and other cereals, and breads contributed to >60% of the new foods added, with vegetable-based dishes alone making 30%.

A performance evaluation of the tool was based on dietary recall data from 29,113 participants with data collected from January 2020 to September 2021 (Figure 1). About 55% of the dietary data was from Sri Lanka and South India, followed by Pakistan, North India, and Bangladesh (Table 1). Mean age of participants in SAB was 46.7 y (SD: 13.9 y). Table 1 shows higher mean age in Sri Lanka and South India with a larger proportion of participants aged 60 y and older. Females constituted >60% of the sample. While participants were from diverse educational and economic backgrounds, those from India and Sri Lanka exhibited a relatively greater proportion of individuals with primary education or higher, along with a higher median income (Table 1).

**TABLE 1:** Characteristics of the South Asia Biobank participants with completed 24-h dietary recall data in each region (*N* = 29,113).

	Bangladesh (%)	Pakistan (%)	Sri Lanka (%)	North India (%)	South India (%)	Total (%)
No. of participants	4029 (14)	4922 (17)	9756 (33)	4099 (14)	6307 (22)	29,113
Age (y), mean (SD)	42.5 (13.3)	45.4 (13.6)	47.2 (14.4)	43.5 (13.8)	51.8 (11.9)	46.7 (13.9)
Age groups (y)						
18–39	46.7	33.9	30.7	40.3	15.7	31.6
40–59	39.9	49.5	47.2	45.6	55.8	48.2
60 y and over	13.4	16.7	22.1	14.1	28.4	20.2
Sex (female)	52.6	66.8	68.8	61.4	53.7	61.9
Level of education						
None or below primary	40.2	55.6	11.9	21.5	34.7	29.5
Primary	40.0	9.8	38.4	19.6	24.3	28.1
Secondary	8.6	10.0	32.6	17.4	18.8	20.3
Higher than secondary	11.2	24.6	17.2	41.6	22.2	22.1
Occupation (in paid employment)	45.4	28.2	43.0	42.0	54.6	43.2
Income USD/PPP, median (IQR)	450 (300–600)	400 (300–600)	560 (300–800)	800 (480–1200)	600 (400–800)	540 (330–800)

Dietary data collected from January 2020 to September 2021; excluded participants with sex other due to small number (*n* = 17). Income was reported in US dollar after being adjusted for purchasing power parity (PPP) for comparability between regions.

Table 2 shows key findings from the QC checks. Median recall completion time was the lowest in South India (10 min) and highest in Bangladesh (22 minutes). In total, 24% of the recalls were completed in 10 min or less, with about half of these coming from South India. One percent of the total recalls had fewer than 8 items recorded, and 8% had recorded missing foods. On average, implausible energy intake was observed in 4% of the recalls: under-reporting (energy <500 kcal/d in females and <800 kcal/d in males) was seen in 2% of the recalls, with the highest contributions from South India and Pakistan. Similarly, over-reporting (energy >3500 kcal/d in females and >4200 kcal/d in males) was observed in 2% of the total recalls, with the highest in Bangladesh.

**TABLE 2:** Tool performance evaluation checks conducted on 24-h dietary recalls collected using the South Asia version of Intake24 (*N* = 29,113).

Quality control checks		Bangladesh	Pakistan	Sri Lanka	North India	South India	Total Sample
No. of recalls	*n*	4029	4922	9756	4099	6307	29,113
	%	14	17	33	14	22	100
Recall completion time (min)	Mean	27	14	15	16	16	17
	SD	51	43	10	27	45	36
	Median	22	12	14	12	10	13
	IQR	18–28	11–14	11–17	9–17	7–15	10–18
Completion time ≤10 min	*n*	194	700	1684	1289	3158	7025
	%	5	14	17	31	50	24
Completion time ≤5 min	*n*	7	19	4	104	363	497
	%	0	0	0	3	6	2
Completion time ≤2 min	*n*	0	0	0	0	2	2
	%	0	0	0	0	0	0
Recalls with fewer than 8 items^1^	*n*	11	47	52	53	17	180
	%	0	1	1	1	0	1
Recalls with missing foods	*n*	248	196	1854	88	52	2438
	%	6	4	19	2	1	8
Recalls by plausible energy intake							
Under-reporting (female <500 kcal/d; male <800 kcal/d)	*n*	31	114	81	38	213	477
	%	1	2	1	1	3	2
Over-reporting (female >3500 kcal/d; male >4200 kcal/d)	*n*	143	34	249	113	13	552
	%	4	1	3	3	0	2

Dietary data collected from January 2020 to September 2021; excluded participants with sex other due to small number (*n* = 17).

1 Excluding associated foods, that is, foods that are commonly consumed together, for example, milk and sugar with tea and butter with toast.

**FIGURE 6:**
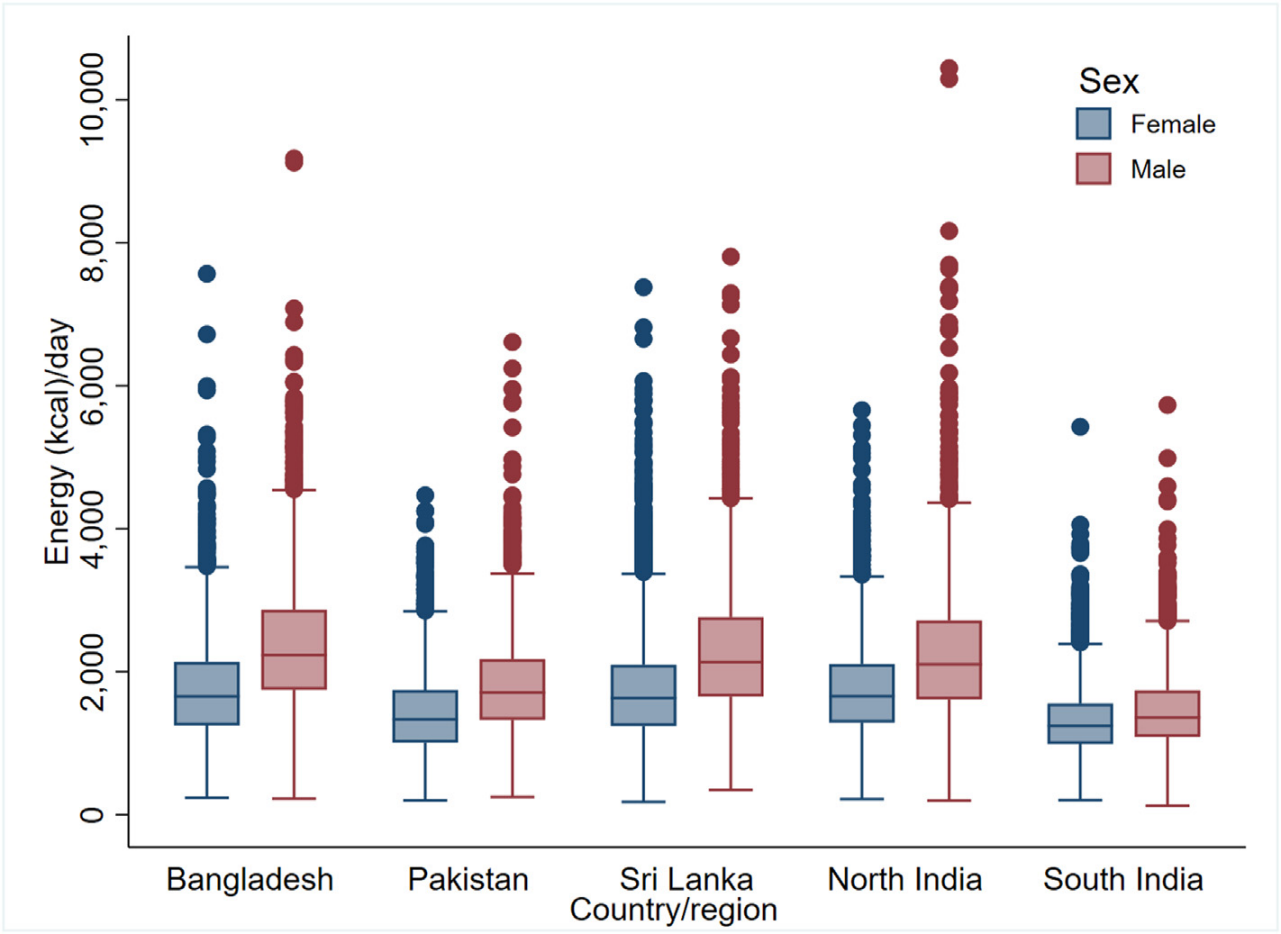
Energy box plots by sex and region (*n* = 29,113). Outliers were detected as values outside the range defined using 3 times the IQR from the nearest quartile.

**FIGURE 7:**
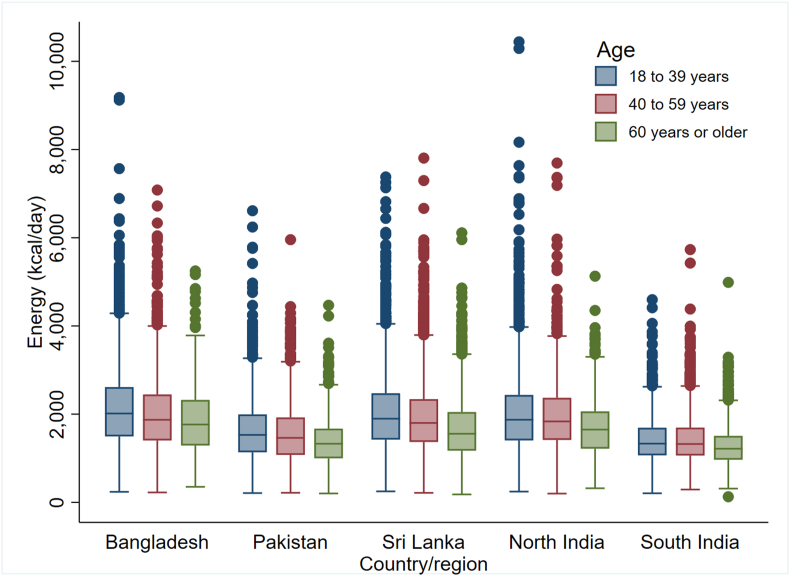
Energy box plots by age and region (*n* = 29,113). Outliers were detected as values outside the range defined using 3 times the IQR from the nearest quartile.

**FIGURE 8:**
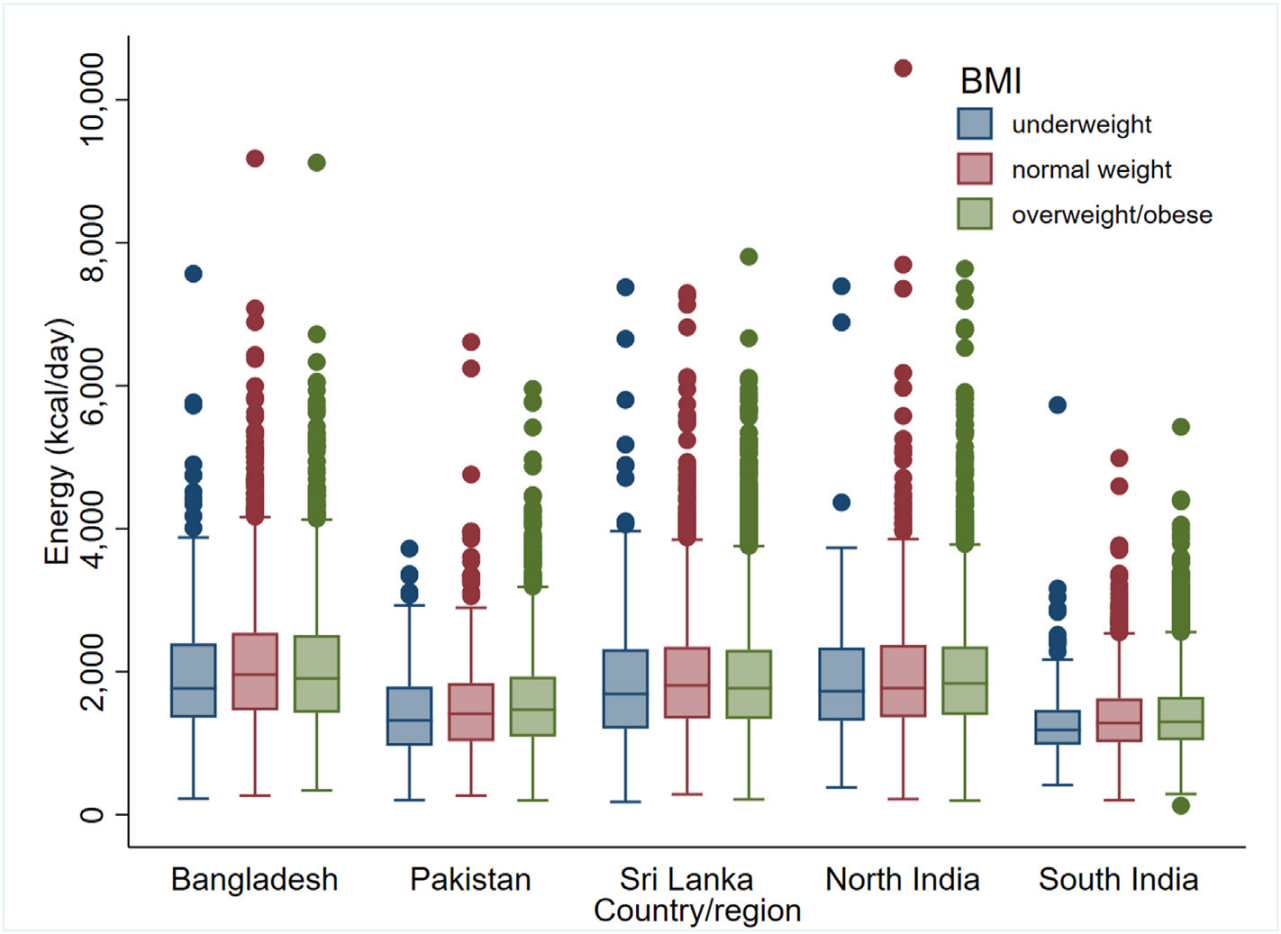
Energy box plots by BMI (*n* = 28,793; 320 recalls with missing values for BMI were not included). Outliers were detected as values outside the range defined using 3 times the IQR from the nearest quartile. Note: South Asian population specific BMI cut-points were used (see Methods section).

Energy box plots showed a consistent trend across all SAB regions: higher median energy intake among younger individuals compared to older age groups and in males compared to females (FIGURE 6, FIGURE 7). Notably, underweight participants had lower estimated energy intake, with no discernible differences in energy intake observed across other BMI categories (Figure 8).

Portion size box plots (data not shown) showed tool-related errors contributing to unusually high portions for 3 key foods. Specifically, 3 errors were identified and corrected, informed by consultation with regional teams and literature review: *1*) for Sri Lankan sweet snack string hoppers, implausibly high portions were winsorized at 720 g; *2*) in Intake24, small fried fishes were incorrectly assigned 100 g portions instead of 10 g, and so reported amounts were divided by 10; and *3*) local breads like chapatti/roti and paratha were assigned high portion sizes in Intake24, which resulted in local breads being overestimated in all regions except Pakistan. Following consultation with regional teams and review of portion sizes for local breads, as corrective action, we reduced recorded portions by 50% in all regions but Pakistan. These data adjustments and tool corrections resulting from the box plot investigations were a valuable step toward data preparation and subsequent improvements to the tool.

## Discussion

South Asia has an extensive and diverse food supply and a rich variety of food preparations, eating behaviors, and cultures and it is therefore crucial that dietary instruments reflect the diversity of foods and consumption patterns of South Asian populations. We have described the adaptation, implementation, and evaluation of Intake24 for dietary data collection in adults living in 5 regions of South Asia. To our knowledge, our adaptation is the first automated digital tool for standardized interviewer-administered 24-h recalls, quantifying dietary intake in South Asians.

Regional teams contributed to various aspects of tool adaptation and implementation including selection of foods for the South Asian food database, identification of appropriate portion estimation methods, and the collection of dietary recall data. The tool was further refined in response to feedback received during interviewer training sessions (e.g. addition and refinement of descriptions of local foods, inclusion or exclusion of food prompts, and updates to portion estimation methods). Currently (at the time of publication), the South Asia version of Intake24 contains 2283 foods with linked portion estimation methods, food prompts, and energy and nutrient composition data. Our performance evaluation of the South Asia version of Intake24 in ∼30,000 SAB participants showed that the tool provides good coverage of foods consumed across SAB regions, as indicated by the small proportion of recalls with reported missing items.

Quality aspects of dietary recalls such as recall completion time of 10 min or less and fewer than 8 items reported are possible indicators of incomplete reporting. Recalls completed too quickly or those with fewer items may be associated with under-reporting of food consumption, which could lead to lower energy and nutrient estimates. Using scatter plots, we visually assessed the relationship between energy intake and recall completion time and found no association (data not shown). Although only 2% of total recalls were suggestive of under-reporting based on the prespecified criteria, validation of reported energy intake using objective measures was not undertaken. The relationship between estimated energy intake and age and sex in all SAB regions was in the direction expected from the extensive existing knowledge in most populations—that is, lower energy intake in females as compared to males and higher energy in younger as compared to older participants [32]. We found little difference in energy intake by BMI status except that it was lower in those who were underweight. This may have possibly resulted from a phenomenon seen in dietary data called the flat-slope syndrome and defined as an overestimation of portion sizes in those who eat smaller portions and an underestimation of portion sizes by those who eat larger portions [33].

### Strengths and limitations

A key strength of the adapted Intake24 tool configured for South Asia is that it is the first automated digital 24-h dietary recall tool designed for use in South Asia across different countries and regions. Our incorporation of regional and local foods from each of the 5 SAB regions into the tool’s food database enabled the implementation of a standardized single tool that facilitates cross-country comparisons. The interviewer-led fieldwork design in SAB enabled dietary data collection from participants with a wide range of educational and economic backgrounds, reducing bias from literacy or digital competency-based completion of the tool had it been self-administered. Use of Intake24 enabled the assessment of detailed quantitative dietary intake data with less time and resource burden as compared to traditional pen-and-paper approaches, which subsequently require manual data coding.

This report provides an assessment of the tool’s performance based on the first SAB data release (including dietary data collected from January 2020 to September 2021) including ∼30,000 participants with dietary data. Cleaning and preparation of the data collected subsequently is underway. Once completed, the study will be in a unique position to examine the regional distributions and determinants of dietary intakes across food groups, nutrients, and dietary patterns as well as the associations between dietary factors and cardiometabolic disease risk in a large sample of South Asians.

Intake24 adaptation had limitations. Portion sizes for some South Asian foods were adapted from comparable items within the UK Intake24, and these may not fully represent the portion sizes consumed in South Asia. Foods were matched pragmatically to UK food composition values to derive nutrient content using a “best basic” match approach, and this may not well reflect recipes of local South Asian foods, dishes, and manufactured products. Variations in nutrient composition of foods across SAB regions, arising from differences in ingredients and cooking methods are also unlikely to be fully captured. However, further work to refine the food composition information for more accurate nutrient representation of regional foods is underway as phase 2 of the project, as previously mentioned. Since the South Asia version of Intake24 was configured in English, recall administration relied on the interviewers understanding of English and subsequent translation to regional languages as required for data collection from participants. This may have impacted data collection due to potential differences in question wording, food names, and descriptions during translation. Moreover, logistical issues such as busy study sites with multiple participant measurements to be completed may have affected dietary data collection.

We were able to collect dietary data only for a single day from each participant. This was due to resource constraints and risk of participant burden. External factors such as the COVID-19 pandemic and related economic and societal hardship also affected the feasibility of repeated data collection. A single 24-h dietary recall is subject to within-person variation, which cannot be quantified or adjusted for without repeated recalls. However, it does allow for detailed ascertainment of actual foods and drinks consumed with the ability to derive absolute consumption on a single day. With appropriate acknowledgment of the limitations of a single recall, the dietary data collected can provide useful estimates of average dietary intakes over a 24-h time frame in a large sample across 5 regions of South Asia. Lack of validation of the Intake24 tool in regions of South Asia against another method in this study is a limitation, though the performance evaluation of the tool yielded findings in the expected direction for key parameters including energy intake differences by sex and age. A validation of reported energy intake from the Intake24 tool against both subjective and objective measures of energy expenditure is currently underway. This ongoing work aims to strengthen the accuracy of the adapted Intake24 tool for dietary assessment in South Asian populations.

The adaptation of Intake24 for use in the SAB has provided valuable insights into the development of automated dietary assessment tools for diverse populations. This adaptation was critically informed through collaboration with local teams, particularly those with nutrition expertise, to create a comprehensive food database of the list of foods included in the tool that provides cultural relevance and minimizes missing food items. Incorporating local food names, culturally appropriate food images, and prompts tailored to regional dietary habits enhanced the usability and acceptability of the tool. Enabling hybrid data collection methods (online and offline) was crucial to ensure reliable dietary data capture, especially in areas with limited internet connectivity. Regular monitoring and addition of frequently reported missing foods kept the tool updated with diverse regional foods. Maintaining an up-to-date food database will be critical as new food products enter the market. Furthermore, participants experienced time constraints due to multiple assessments during their study visits. Researchers can consider streamlining on-site workflows to optimize the time available for dietary data collection. Confidence in the Intake24 dietary assessment tool would be improved by further evaluation, including potentially a usability assessment in regions of South Asia. Dietary data quality can be improved by updates to the underpinning food composition information, particularly to ensure recipes appropriately reflect local dishes. Assessment of dietary and nutritional intakes in the SAB would also be substantially improved by the collection of >1 recall in a subset of participants, which would allow for data adjustments to better estimate usual intakes.

### Conclusions

We described the adaptation of Intake24, an existing digital multiple pass 24-h dietary recall tool from the United Kingdom, for dietary assessment in South Asia and its implementation for the first time across 4 countries of South Asia. Interviewer-led administration of a digital instrument that is contextually tailored to collect dietary data from South Asians has the potential to revolutionize dietary assessment in South Asian populations by enhancing feasibility, cost-effectiveness, and cultural applicability. Application of the adapted Intake24 will enable the description and comparisons of dietary patterns, food and nutrient intakes, evaluation of intakes against recommended guidelines, and the future investigation of the link between dietary risk factors and health end points in South Asians living within its different regions. Intake24 is open-source and freely available for use by researchers wishing to collect dietary intake data from South Asians.

## Author contributions

The authors’ responsibilities were as follows – JC, RMA, KIK, VJ, AK, PK, MKM: are investigators of the South Asia Biobank responsible for the overall study design; DB, BH, BA, TS, BR, SA, PP: undertook the adaptation of Intake24, with scientific input from NGF; RP, SM, PM, LS, LA, AAS: provided input for Intake24 adaptation; BH, BR, DB: trained interviewers to conduct 24-h dietary recalls; DB, DC, BA, SA: analyzed the dietary data with inputs from TS, PP, and NGF; DB: wrote the first draft of the manuscript; TS, PP, NGF: gave inputs to the first draft; and all authors: read and approved the final manuscript.

## Data availability

The South Asia Biobank data are available to researchers upon request. Data requests should be made via email to the study Steering Committee (john.chambers@imperial.ac.uk). Intake24 is open source and available to use (support@intake24.org).

## Funding

The South Asia Biobank is funded by the National Institute for Health Research (NIHR) Global Health Research Unit on Diabetes and Cardiovascular Disease in South Asia (16/136/68 and 132960). The views expressed in this publication are those of the author(s) and not necessarily those of the NIHR or the Department of Health and Social Care. The funder had no role in study design, data collection and analysis, decision to publish, or preparation of the manuscript. J.C. is supported by the Singapore Ministry of Health’s National Medical Research Council under its Singapore Translational Research Investigator (STaR) Award (NMRC/STaR/0028/2017). NGF acknowledges core support from the Medical Research Council Epidemiology Unit (MC UU 00006/3). DB, NGF, and PP acknowledge funding from the NIHR Cambridge Biomedical Research Centre (203312) and NGF is an NIHR Senior Investigator (NIHR202397).

## Conflict of interest

The authors report no conflict of interest.
