# Supplementary material for: The Adaptation, Implementation, and Performance Evaluation of Intake24, a Digital 24-h Dietary Recall Tool for South Asian Populations: The South Asia Biobank
Source: Curr Dev Nutr. 2025 Jan 16;9(2):104543. doi: 10.1016/j.cdnut.2025.104543 (PMC11847516; doi:10.1016/j.cdnut.2025.104543)
Supplement: Multimedia component 1 [file mmc1.docx]

**Supplementary Table 1**: Examples of region-specific foods added to the South Asia version of Intake24.

| Country / region | Examples of region-specific foods added to the South Asia version of Intake24 |
| --- | --- |
| **Bangladesh** | Rice puffs, with peanuts & deep-fried chickpea flour noodles (e.g. Haldiram's) (Muri/ Mudhi / Murmura / Kurmura)  Sweet dessert, roasted vermicelli noodles, milk, cream, and sugar (Dudh Shemai)  Dried fish curry (Sutki macher tarkari/jhol)  Potato fried with Kaski fish (Alu-Kaski vaji) |
| **Pakistan** | Mixed lentil with wheat, barley, and shredded mutton (Haleem/ Daleem)  Trotters, cooked (Paya)  Naan, made with butter/oil/ghee and sesame seeds (Roghni Naan)  Lamb and potato curry (Aloo gosht) |
| **Sri Lanka** | Coconut roti, made with rice flour and coconut (Pol roti)  Rice flour and coconut milk crepe, plain (Plain hopper/ Appa / Appam)  Jackfruit curry, with coconut milk (Kiri kos)  Coconut custard pudding, with coconut milk, jaggery, cashew nuts, egg, and spices (Watalappan) |
| **North India** | Paratha, made with white chapati flour and stuffed with potato (Aloo paratha)  Paneer and bell pepper curry (Kadhai paneer)  Black lentil dahl, with butter and cream (Dal makhani / Black dal)  Sweet dessert, black gram or maida flour pretzels, deep-fried, in sugar syrup (Jalebi) |
| **South India** | Savoury cake, with rice, steamed (Idli)  Rice and black gram dahl crepe, fermented batter (Dosa / Dosai / Thosai, plain)  Soup, with tamarind juice or pulp and tomato (Rasam)  Sweet milk pudding, with green gram (Green gram kheer/ Payasam green gram / Paruppu payasam/ Hesaru bele paysam) |
